# Do Modic changes contribute to lumbar instability or other way around? A retrospective study based on their types, extents, and affected lumbar segments

**DOI:** 10.1186/s12891-023-07011-7

**Published:** 2023-11-11

**Authors:** Xiaoping Mu, Hansheng Deng, Xiaodong Wei, Jianxun Wei, Gianfilippo Caggiari

**Affiliations:** 1 Department of Spine Surgery, The People’s Hospital of Guangxi Zhuang Autonomous Region, Guangxi Academy of Medical Sciences, No.6, Taoyuan Road, Nanning, 530021 China; 2 Department of Biomedical Sciences, University of Sassari, 07100 Sassari, Italy; 3 Orthopaedic Department, Sassari University Hospital, 07100 Sassari, Italy; 4 Shenzhen Children’s Hospital of Shantou Medical University, Shenzhen, People’s Republic of China

**Keywords:** Chronic low back pain, Modic changes, Lumbar segmental motion, Angular, Translation, Lumbar instability

## Abstract

**Background:**

Which types of Modic changes (MCs) and whether or how specific factors associated to MCs work on lumbar instability have yet to be well understood. The purpose of this study was to investigate the influences of the types of MCs, the extent of MCs lesion involvement, and different lumbar levels involved by MCs on lumbar instability.

**Methods:**

This retrospective study included 263 adult subjects with MCs who underwent lumbar X-ray examinations in the neutral, flexion, and extension positions. All patients who met our inclusion criteria were examined with 1.5 Tesla magnetic resonance units. Two experienced authors with more than three-year clinical experience independently evaluated and measured the subjects’ radiographic images. The subgroup analysis was performed to detect the differences in subjects’ baseline characteristics and lumbar segmental motions among three types of MCs, the extent of MCs lesion involvement and different lumbar levels involved by MCs.

**Results:**

There was a statistical difference in body mass index (BMI) between different involvement extent of MCs (*p* < 0.01), indicating that the subjects with high BMI are more likely to develop severe MCs. The subjects with Modic type 1 change (MC1) had a significant increase in lumbar angular motion than those with Modic type 2 change (MC2) and Modic type 3 change (MC3) (*p* < 0.01) and compared with MC3, a significant increase in lumbar translation motion was detected in subjects with MC1 and MC2 (*p* < 0.01). While, angular motion decreased, translation motion increased significantly as the extent of MCs lesion involvement aggravated (*p* < 0.01). However, there were no statistical differences in lumbar angular and translation motions between different lumbar levels involved by MCs (*p* > 0.05).

**Conclusions:**

Higher BMI might be a risk factor for the development of severe MCs. MC1 and MC2 significantly contribute to lumbar instability. The extents of MCs lesion involvement are strongly associated with lumbar instability. However, different lumbar levels involved by MCs have little effect on lumbar stability.

## Background

Chronic low back pain (CLBP) was defined as low back pain for more than three months [1], which is the most common musculoskeletal disorders that affects over 80% of individuals at least once in their lifetime [2]. CLBP results in substantial morbidity but its etiology and pathophysiology remain unclear [3]. The structural abnormalities of the spine are well recognized as causes of CLBP [3].

Signal changes of vertebral bone marrow adjacent to the endplate represent a specific type of lesion, also known as Modic changes (MCs) [4, 5], which have been demonstrated to be closely correlated with CLBP in communities-based populations [6, 7] and clinical patients [8–10]. Lumbar biomechanics and local inflammatory might be able to response well to the source of CLBP in populations with MCs [11, 12]. Despite it is unclear why and how the initial Modic type 1 change (MC1) occurs, its pathological manifestations indicates a close association with local inflammation [4]. Local inflammation could lead to biomechanical changes in the lumbar spine, which may have some impact on lumbar stability. Moreover, MCs are always related to severe disc degeneration and micro-fracture [11]. Structural abnormalities of the lumbar spine also significantly contribute to lumbar instability.

Several studies [10, 13] have focused on investigating the relationship between MCs and lumbar segmental instability, reporting that MC1 is closely correlated to lumbar instability. However, Modic type 2 change (MC2) seems not to be often stable [11, 13]. The contributions of MC2 and Modic type 3 change (MC3) to lumbar instability have not been well understood. Moreover, few studies have been conducted to explore whether or how certain factors associated to MCs work on lumbar instability.

Therefore, the purpose of the present work was to evaluate the differences in baseline characteristics between three types of MCs and between the different extents of MCs lesion involvement, as well as investigate whether the types of MCs, the extent of MCs lesion involvement, and different lumbar levels involved by MCs contribute substantially to lumbar instability.

## Methods

### Study population

Electronic medical record was retrospectively reviewed for all subjects with MCs at a single institution from September 2018 to October 2022. This study included a total of 263 subjects who met the following inclusion criteria: 1) adult subjects (≥ 18 years of age) on admission; 2) underwent lumbar X-ray examination in the neutral (0°), flexion (40°), and extension positions (-20°) and lumbar magnetic resonance imaging (MRI) scanning in the supine position; 3) MRI scanning revealed at least one of the three types of MCs at lumbar level; 4) available and clear images.

Subjects with spinal scoliosis more severe than 15 degrees, or a prior history of lumbar spine, or lumbar specific and/or nonspecific infectious, spinal trauma, and spinal tumor were excluded in this study. Subjects with hip or knee disorders, were also excluded due to substantial impact on gait and posture, which may lead to compensatory changes in lumbar spine.

The selection of the study population and the measurement of imaging parameters were carried out independently by two groups of this study’s authors. The study subjects were sequentially coded and their personal information was covered before measurement. The present work was implemented under the guide of the principles of the Declaration of Helsinki. Patients who participated in this study signed the informed consent provided, declaring they were aware of the study details. The ethical committee of the People’s Hospital of Guangxi Zhuang Autonomous Region, Nanning, People’s Republic of China, reviewed and approved the study protocol (No. 2016–12).

### Radiographic evaluation and measurement

The types of MCs, the involvement extents of MCs, and lumbar segmental motions were independently evaluated and measured by two experienced authors. Two spinal surgeons with more than three-year clinical experience independently evaluated the patients’ MRI.

#### Measurement of lumbar segmental motions

**Fig. 1:**
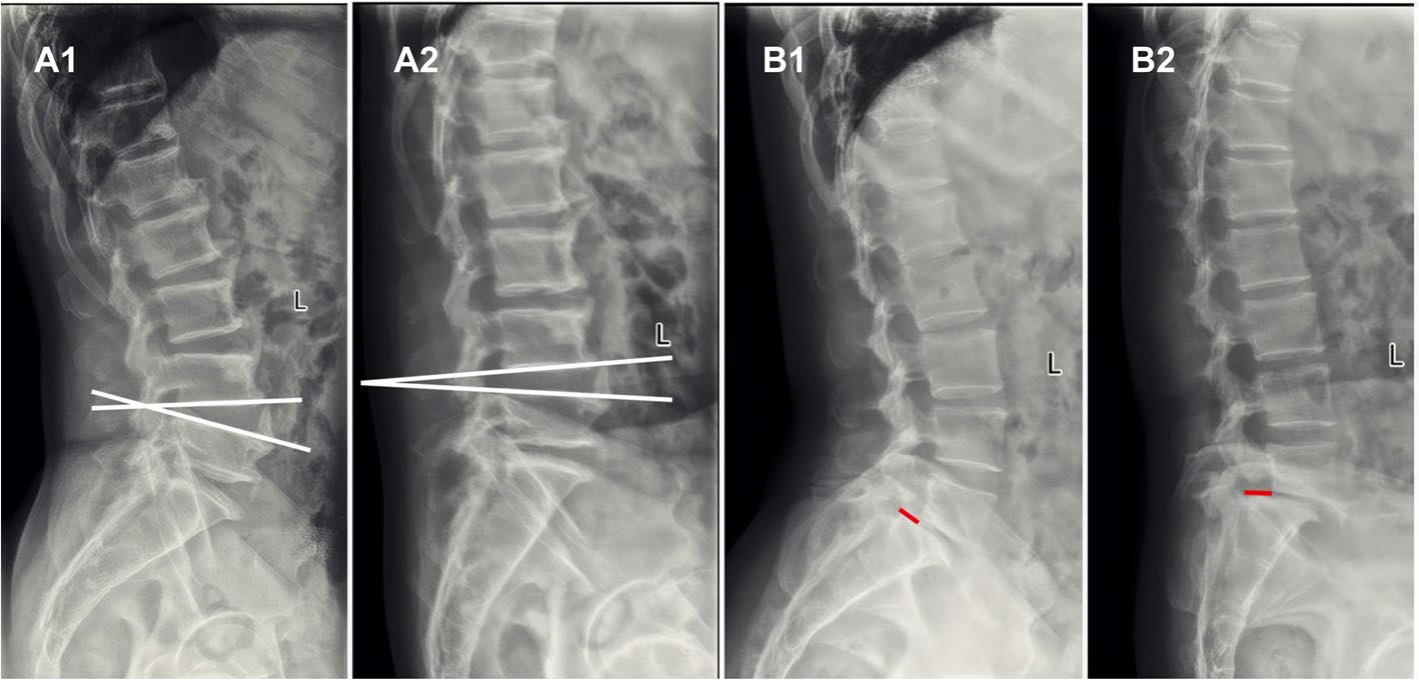
Measurement of angular and translation motions in the flexion and extension positions. Angular motion (**A1, A2**): intervertebral angles in the flexion (**A1**) and extension (**A2**) positions. Translation motion (**B1, B2**): horizontal displacements in the flexion (**B1**) and extension (**B2**) positions

We assessed the segmental angular and translation motions in the flexion and extension positions on X-ray images (Fig. 1). Lumbar vertebral bodies of the subject from the subjects were marked with four points (anterosuperior, anteroinferior, posterosuperior, and posteroinferior). The angular motion was counted as an absolute value of the difference between the intervertebral angles from flexion to extension. The translation motion was measured as an absolute value of the difference in the horizontal movement of the superior endplates on their adjacent inferior endplates from flexion and extension.

#### MRI equipment parameters

All patients who met our inclusion criteria were examined with 1.5 Tesla magnetic resonance units (Espree, Siemens, Germany). The scanning range was settled from T12 to sacrum 1.

The following parameters were applied: the sagittal T1-weighted imaging (T1WI) [slice thickness (ST): 4 mm, time of repetition (TR): 400 ms, time to echo (TE): 8 ms, field of view (FOV): 280 mm], the sagittal T2-weighted images (T2WI) [ST: 4 mm, TR: 3000 ms, TE: 100 ms, FOV: 280 mm]; and the axial T2WI (ST: 4 mm, TR: 5000 ms, TE: 97 ms, FOV: 250 mm).

### Evaluation of the three types of MCs

**Fig. 2:**
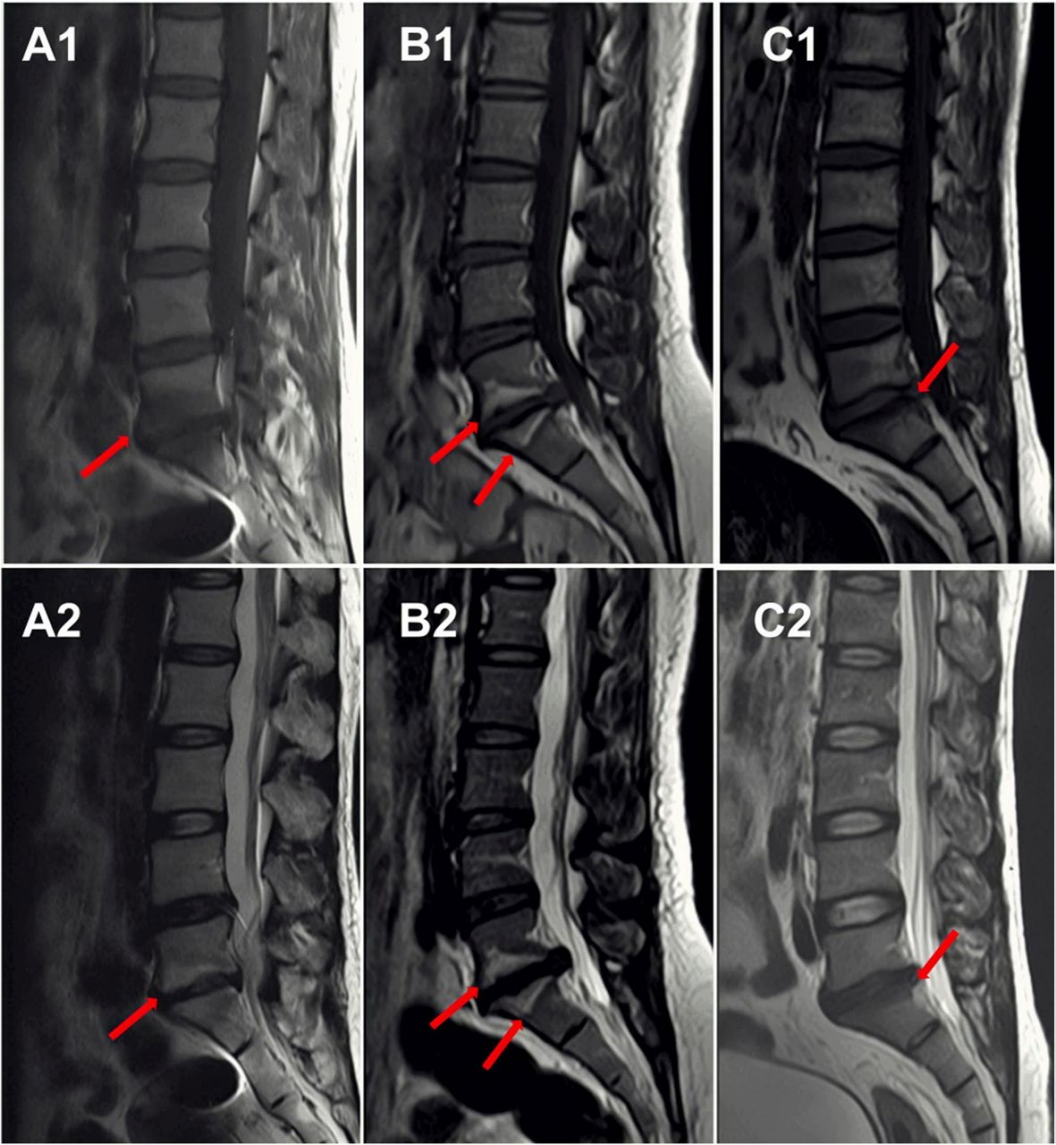
Appearances of three types of MCs. Modic type 1 change (**A1, A2**): Hypointensity on T1-weighted images (T1WI, **A1**) and hyperintensity on T2-weighted images (T2WI, **A2**); Modic type 2 change (**B1, B2**): Hyperintensity both on T1WI (**B1**) and T2WI (**B2**); Modic type 3 change (**C1, C2**): Hypointensity both on T1WI (**C1**) and T2WI (**C2**)

Modic et al*.* [4, 5] classified MCs into three types according to their imaging appearance on T1WI and T2WI (Fig. 2). MC1 shows a decreased signal intensity on T1WI but an increased signal intensity on T2WI. MC2 demonstrates the increased signal intensity both on T1-and T2WIs. In contrast, MC3 reflects the decreased signal intensity both on T1- and T2WIs.

### Definition of the sizes of MCs

According to the classification proposed by Hanımoğlu et al. [8], the vertebral body and endplate are equally divided into 4 parts horizontally and vertically, comprising of 16 small cells. Each small cell involved by MCs is recorded and summed up to calculate the total involvement area of MCs for this subject. I, II, III, and IV represent < 25%, 25%-50%, 50%-75%, and > 75% of the endplate involved by MCs in the horizontal plane, respectively. 1, 2, 3, and 4 represent < 25%, 25%-50%, 50%-75%, and > 75% of the vertebral body involved by MCs in the vertical plane, respectively. Therefore, I1 indicates that MCs invade a small cell, which involve less than 25% of vertebral body and endplate both in horizontal and vertical directions; I2 means that two small grids were involved by MCs, which invade less than 25% of vertebral endplate in the horizontal plane but 25%-50% of the vertebral body in the vertical plane and so on. We define the involvement area of MCs < 25% of the vertebral body in the horizontal or vertical plane as slight, 25%-75% as moderate, > 75% as severe.

### Inter- and intra-observer reliability

We randomly selected 15 of all included subjects to independently evaluate the types of MCs, the extent of MCs lesion involvement, and lumbar segmental motions by two authors (M.X.P. and X.D.W.). The above-mentioned radiographic evaluation and parameters were requested to be repeated 10 days after their first evaluation. The inter- and intra-observer reliability was calculated after they evaluation. An intraclass correlation coefficient (ICC) value > 0.80 represented perfect agreement, 0.61–0.80 substantial agreement. It should be considered unreliable if an ICC value less than 0.60 [14].

### Statistical analysis

We performed the data analysis using IBM SPSS 22.0 (SPSS Inc., Armonk, New York) in the present work. Mean ± standard deviation (SD) was calculated to manifest the continuous variables. We adopted one-way variance analysis or Kruskal–Wallis test to analyze the differences in subjects’ age, BMI and their lumbar segmental motions between the groups. For dichotomous variables such as subjects’ gender, chi-square test was used in this study. A *p* ≤ 0.05 was considered as a significant difference. Intra-class correlation coefficient (ICC) was employed to calculate the inter- and intra-observer reliability in the extent of MCs lesion involvement, lumbar angular and translation motions. Cohen's coefficient kappa was used to identify the inter- and intra-observer reliability in the types of MCs,

## Results

### Inter- and intra-observer reliability

The ICC and kappa values of the inter- and intra-observer reliability in the types of MCs and the image parameters were more than 0.80, indicating that the authors agreed on MCs’ classification and there was an accordance of image interpretation.

### Baseline characteristics

This study included a total of 263 subjects (150 females and 113 males), with a mean age of 57.0 years old and a mean body mass index (BMI) of 22.7 kg/m^2^. Baseline characteristics among three types of MCs and the different extents of MCs lesion involvement are shown in Table 1.

**Table 1:** Baseline characteristics among three types of Modic changes (MCs) and different extents of MCs lesion involvement

Subgroups	Age (years)	Gender (female %)	Body mass index (kg/m^2^)
Three types of Modic changes (MCs)			
MC1 (*n* = 89)	57.5 ± 10.5	44 (49.4%)	22.7 ± 3.2
MC2 (*n* = 131)	56.0 ± 10.3	73 (55.7%)	22.6 ± 2.8
MC3 (*n* = 43)	58.2 ± 8.3	28 (65%)	22.8 ± 3.2
*p*	n.s	n.s	n.s
Involvement extents of MCs			
Slight (*n* = 113)	57.6 ± 10.0	62 (54.9%)	21.7 ± 2.7^*^
Moderate (*n* = 82)	56.0 ± 9.8	50 (61.0%)	22.8 ± 2.8^*^
Severe (*n* = 68)	57.3 ± 10.0	38 (55.9%)	24.3 ± 3.2
*p*	n.s	n.s	< 0.01

mean ± SD*; n.s*. no significance

^*^compared with group of severe, *P* < 0.05

According to the types of MCs, 263 subjects were divided into three groups: 89 subjects with MC1, 131 with MC2, and 43 with MC3. The statistical analysis revealed no significant differences in subjects’ baseline characteristics including age, gender, and BMI between three types of MCs (*p* > 0.05).

43.0% of subjects (113/263) were graded as slight, 31.1% (82/263) as moderate, and 25.9% (68/263) as severe based on the grading system of the extent of MCs lesion involvement. There were no significant differences in age and gender among the three groups (*p* > 0.05). However, a statistical difference in BMI was detected between the different extents of MCs lesion involvement (*p* < 0.01).

### Lumbar segmental motions

Lumbar angular and translation motions in patients with different types of MCs, the different extents of MCs lesion involvement, and different lumbar levels involved by MCs are shown in Table 2.

**Table 2:** Lumbar segmental motions in patients with different types of Modic changes (MCs), different involvement extents of MCs, and different lumbar levels involved by MCs

Subroups	Angular motion (degrees)	Translation motion (degrees)
Three types of Modic changes (MCs)		
MC1 (*n* = 89)	7.3 ± 1.9	2.2 ± 2.0
MC2 (*n* = 131)	6.5 ± 2.1	2.3 ± 2.1
MC3 (*n* = 43)	6.4 ± 1.8	1.4 ± 1.5
*p*	< 0.01	< 0.01
Involvement extents of MCs		
Slight (*n* = 113)	7.5 ± 2.0	1.4 ± 1.6
Moderate (*n* = 82)	6.4 ± 1.9	2.1 ± 1.9
Severe (*n* = 68)	5.9 ± 1.6	3.0 ± 2.2
*p*	< 0.01	< 0.01
Lumbar levels involved by MCs		
Single level (*n* = 189)	6.8 ± 2.0	2.1 ± 2.0
Double levels (*n* = 50)	6.3 ± 1.8	2.3 ± 2.0
Triple levels (*n *= 24)	6.8 ± 1.9	1.5 ± 1.5
*p*	n.s	n.s

mean ± SD; *n.s*. no significance

A significant difference in lumbar angular motion was observed between subjects with MC1 and those with MC2 and MC3 (*p* < 0.01). Compared with MC3, a significant increase in lumbar translation motion was detected in subjects with MC1 and MC2 (*p* < 0.01). Considering the potential influence of the the extent of MCs lesion involvement on lumbar segmental motion, while the subgroup analysis focusing on lumbar angular and translation motions of the different extents of MCs lesion involvement was performed. The statistical analysis indicated that angular motion decreased, translation motion increased significantly as the extent of MCs lesion involvement aggravated (*p* < 0.01). However, there were no statistical differences in lumbar angular and translation motions between different lumbar levels involved by MCs (*p* > 0.05).

## Discussion

### Main findings

The present work revealed that age, gender, and BMI may contribute similarly to the occurrence of three types of MCs. Moreover, subjects with MC2 and MC3 show the significant decreases in angular motion; the significant increases were found in the translation motion of those with MC1 and MC2. Lumbar angular motion decreased, translation motion increased significantly as the the extent of MCs lesion involvement aggravated. However, single or multiple lumbar levels involved by MCs is not strongly associated with lumbar segmental instability.

### Baseline characteristics and MCs

MCs can be observed not only in adults [15] but also in children [16]. However, most studies have reported that the prevalence of MCs increases with age, with a high incident rate in patients over 50 years [12, 17]. The mean age of the 263 patients with MCs included in our study was 57.0 (SD: 9.9) years, consistent with the findings of previous studies. A recent study [18] has demonstrated that young patients are closely associated with MC1. However, we did not detect any significant difference in age among the three types of MCs. The small sample size of the previous study (MC1: 35 cases, MC2: 110 cases) tended to result in lower statistical power and may have contributed to this discrepancy. Further studies with a large size could help to clarify this clinical question.

The association between MCs and gender remains a controversial issue. Men are often engaged in medium and heavy physical work [11] and are prone to endplate microfractures [18, 19], which may account for high incidence of MCs in such the population. However, a plausible explanation to the high prevalence of MCs in women may be the result of osteoporosis generated by a change in hormone levels [11, 19] Although no statistical difference in gender was detected between different types of MCs in the present study, one study [18] has shown that men are associated with MC1, and women are associated with MC2. However, the pathophysiological mechanisms underlying the effect of sex differences on the prevalence of three types of MCs are not well documented by the authors and the existing evidence.

Three types of MCs represent different pathological stages of the disease [20]. Although the BMI is strongly correlated with the occurrence of MCs compared with those without MCs [19], it may be not a key factor affecting the pathological processes of MCs based on the result of this study. However, the mechanical load may play an important role in the development of MCs [21]. MCs always originate in the endplate and progressively advance to the vertebral body if irritant’s presence persists. Population with high BMI are more prone to the endplate and vertebral fatigue microfractures [22], and the fracture gaps gradually serve as a bridge between the intervertebral disc and vertebral body, which provides a prerequisite for the subsequent diffusion of inflammatory mediators and metabolites from the disc to the endplate and vertebral body [19]. This also explains very well why populations with severe MCs have higher BMI in this study.

### Lumbar segmental motion and MCs

Segmental instability is a potential factor in a population with symptomatic MCs, a thorough understanding of the kinematic characteristics of MCs facilitates clinical decision-making and selects the best treatments for physicians [23]. Previous studies [13, 23] have reported that subjects with MC1 showed a significant increase in angular motion, whereas a significant decrease in translation motion tends to be found in subjects with MC2. The results of the present work support most of the findings of the above study. However, compared to subjects with MC3, MC2 also showed a significant increase in translation motion similar to MC1, suggesting that MC1 is closely associated with lumbar instability and MC2 may not be as stable as expected. Angular motion increased in MC1 and decreased in MC2 and MC3 may indicate intervertebral disc degeneration aggravated with types of MCs increased, which coincides with the pathological manifestation of the transformation of three types of MCs, i.e. from the stage of inflammatory and edema (MC1) to the degenerative phase (MC2) or bone sclerosis (MC3) [4, 5].

There are many factors associated with MCs that may contribute to lumbar instability. However, most of them have not been thoroughly studied. At least to the authors’ knowledge, this is the first work to investigate the impacts of three types of MCs, the extent of MCs lesion involvement, and different lumbar levels involved by MCs on lumbar instability. This study did not compare differences in segmental motion between subjects with or without MCs under the same grade of disc degeneration, as Hayashi et al*. *[13] did. However, we confirmed their findings that lumbar segmental motion may be influenced not only by disc degeneration but also by the MCs themselves from the new perspective of the the extent of MCs lesion involvement. In this study, the results revealed that the the extent of MCs lesion involvement positively contribute to lumbar instability. Therefore, it should also be taken into consideration in developing treatment strategies. Additionally, we hypothesized that lumbar levels involved by MCs may be the potential factor to affect lumbar segmental motion. However, there was no statistical difference between the groups. This may be related to the fact that MCs are less likely to involve multi-segmental lumbar spine [24] and the involvement extents of most MCs at the double and triple lumbar levels are slight.

### Limitations

This study bears with certain deficiencies. First, the qualitative method, i.e. slight, moderate, and severe, to grade the the extent of MCs lesion involvement may not be superior to the quantitative method. However, the results generated from this study are very interesting and still highly credible. Second, angular and translation motions could be affected by lumbar level, but this study was not able to analyze the effects of MCs on the segmental motion at each lumbar level. Despite the present work revealed several factors associated with MCs that contribute to lumbar instability, it is essential to explore the correlations between clinical symptoms, management options and these factors.

## Conclusions

The current study reveals that MC1 plays a substantial role in causing lumbar instability. Furthermore, it appears that MC3 has a relatively minor impact on lumbar stability when compared to the other two types of MCs. Interestingly, MC2 does not seem to have as minimal an effect on lumbar stability as previous research has suggested. The degree to which MCs are involved is closely linked to lumbar instability, and a higher BMI may pose a risk factor for the development of more severe MCs. However, the specific lumbar levels affected by MCs have minimal influence on lumbar stability.

## Data Availability

The datasets generated and analyzed during the current study are available from the corresponding author on reasonable request.
